# Editorial: Myelin-Mediated Inhibition of Axonal Regeneration: Past, Present, and Future

**DOI:** 10.3389/fnmol.2017.00113

**Published:** 2017-04-28

**Authors:** Sari Hannila, Wilfredo Mellado

**Affiliations:** ^1^Department of Human Anatomy and Cell Science, College of Medicine, University of ManitobaWinnipeg, MB, Canada; ^2^Laboratory for Axonal and RNA Biology, Burke-Cornell Medical Research InstituteWhite Plains, NY, USA

**Keywords:** myelin inhibitors, myelin associated glycoprotein, Nogo, OMgp, neuronal regeneration

Impact: what every scientist seeks in their career. It is a sign of widespread influence, a reflection of the field's continued interest, and validation of our ideas. The impact of a scientist's work is often expressed using simple numbers found in citation reports, journal impact factors, and h-indices, but perhaps the truest measure is the respect of their peers. By this metric, the impact of Dr. Marie T. Filbin's life and career was profound, as evidenced by the many articles published by friends and colleagues in remembrance of Marie since her passing (Ashe and Roskams, 2014; Maddox, 2014; Melendez-Vasquez, 2014; Roskams et al., 2014; Stephenson, 2014). For those of us who had the privilege of working in her laboratory, our fond personal memories are intertwined with a deep desire to preserve and promote her scientific legacy, and this was the driving force behind this Frontiers Research Topic. There were few things that Marie enjoyed more than talking about science, so we could think of no better way to honor her memory than to invite our colleagues to share perspectives on the current state and future direction of the field she helped create.

The hypothesis that central nervous system (CNS) myelin inhibits axonal regeneration was first proposed in the early 1980s (Berry, 1982), and Marie played a central role in identifying myelin-associated glycoprotein (MAG) as the first myelin-associated inhibitor (McKerracher et al., 1994; Mukhopadhyay et al., 1994). This was soon followed by the discovery of other myelin proteins that inhibit axonal growth, such as Nogo-A (Chen et al., 2000; GrandPré et al., 2000; Prinjha et al., 2000), oligodendrocyte-myelin glycoprotein (Kottis et al., 2002; Wang et al., 2002a), and ephrin-B3 (Benson et al., 2005), as well as the receptors and co-factors that mediate their effects, including Nogo receptor (Fournier et al., 2001; Domeniconi et al., 2002; Oertle et al., 2003), paired immunoglobulin receptor B (Atwal et al., 2008), the p75 neurotrophin receptor (Wang et al., 2002b), and low-density lipoprotein receptor-related protein 1 (Stiles et al., 2013). Since their discovery, these proteins and receptors have been widely investigated as potential targets for promoting axonal regeneration after spinal cord injury.

This e-book begins with several reviews that expand on the theme of myelin and its function in the injured CNS. While myelin-associated inhibitors are typically viewed in a negative light, these proteins do have normal physiologic roles that have been discussed by Baldwin and Giger. The next chapter is a historical and personal overview of the myelin-associated inhibitor field by McKerracher and Rosen. It is followed by a review from Soheila Karimi-Abdolrezaee's lab that describes the pathophysiologic events that affect oligodendrocytes after CNS injury, as well as cellular approaches currently used to promote remyelination (Alizadeh et al.). This topic is examined from a neuronal perspective by Kaplan et al. in their discussion of how neuron-intrinsic factors contribute to both axonal regeneration and inhibitory factor neutralization in the extracellular environment (Kaplan et al.), which serves as a fitting counterpart to the review by Rao and Pearse that comprehensively describes how specific axon-glia signaling pathways influence both myelination and axonal regeneration.

In 1999, the Filbin lab made another seminal contribution to the axonal regeneration field when they reported that elevation of cyclic AMP (cAMP) in postnatal rat neurons was sufficient to overcome inhibition by MAG and CNS myelin (Cai et al., 1999). They subsequently showed that the increase in cAMP enhanced protein kinase A activity and downstream activation of transcription factors such as cAMP-responsive element binding protein (CREB; Gao et al., 2004). To identify which genes were upregulated in response to cAMP, Jason Carmel performed a microarray analysis of cAMP-treated neurons plated on myelin substrates, and together with Wise Young and Ronald Hart, he describes his findings in this collection of reviews (Carmel et al.). Several genes identified in this screen—arginase I, interleukin 6, secretory leukocyte protease inhibitor, and metallothionein I/II—were shown to overcome MAG inhibition in their own right, and their effects and mechanisms are discussed by Siddiq and Hannila.

Interestingly, analysis of the promoter regions of some of these genes revealed that they did not contain cAMP response elements, which suggests that other transcription factors are involved in reversing the effects of myelin-associated inhibitors. Using embryonic mouse neurons grown in the presence of MAG, it was recently demonstrated that another transcription factor, activator protein 1, functions synergistically with CREB to induce arginase I expression (Ma et al., 2014). An earlier study identified several compounds that allowed cerebellar neurons to overcome myelin inhibition, but surprisingly, they did not elevate cAMP (Usher et al., 2010). This led to the hypothesis that non-cAMP-regulated genes also play a substantial role in blocking myelin-mediated inhibition, and it raises the interesting question of how to identify and manipulate regeneration-associated genes to enhance axonal regeneration, a topic that is discussed by Ma and Willis.

Fittingly, we end with a contribution from Marie's laboratory on the subject that first brought her to prominence: MAG biochemistry and function. As a member of the Siglec family, it is well known that MAG can bind complex gangliosides such as GT1b and GD1a (Kelm et al., 1994; Vinson et al., 2001), but the role of sialic acid-binding in MAG-mediated inhibition of neurite outgrowth remains contentious. Najat Al-Bashir's review presents a new working model describing how sialic acid binding at Arg 118 is required to mediate inhibition by soluble but not membrane-bound forms of MAG (Al-Bashir et al.). While this story would appear to bring Marie's career full circle, it is in fact a reminder of all that remains unknown in this field and the work that still lies ahead. New discoveries regarding the effects of MAG, Nogo, and other myelin-associated inhibitors will undoubtedly provoke debate, and that is something Marie would have welcomed.

She also would have been deeply grateful to all of the distinguished scientists who contributed to this Research Topic, and we extend our deepest thanks to each of them for their time and efforts. We also would like to thank the reviewers for their input and the staff of *Frontiers in Molecular Neuroscience* for their support and guidance throughout this process, one that has been personally and scientifically rewarding for both of us.

## Author contributions

All authors listed, have made substantial, direct and intellectual contribution to the work, and approved it for publication.

### Conflict of interest statement

The authors declare that the research was conducted in the absence of any commercial or financial relationships that could be construed as a potential conflict of interest.
